# Thermoresponsive Solid Dispersion to Enhance Dissolution Profile of Atorvastatin Calcium: An Industrially Sustainable Alternative to Conventional Approaches

**DOI:** 10.3390/pharmaceutics18080993

**Published:** 2026-08-11

**Authors:** Abdelrahman Y. Sherif, Mohamed A. Ibrahim

**Affiliations:** Department of Pharmaceutics, College of Pharmacy, King Saud University, Riyadh 11451, Saudi Arabia; mhamoudah@ksu.edu.sa

**Keywords:** thermoresponsive solid dispersion, Pluronic F-68, atorvastatin calcium, Stat-Ease 360, industrial friendliness

## Abstract

**Background**: Poor aqueous solubility of therapeutic molecules remains a limitation in the pharmaceutical development of lipophilic drugs. This requires formulation approaches that provide the desired therapeutic efficacy while being industry-friendly. This study aimed to optimize a thermoresponsive solid dispersion containing atorvastatin calcium to enhance its dissolution performance. **Methods**: Various nonaqueous solvents were screened to select a thermo-modulating agent. A central composite design was employed to investigate the impact of Pluronic F-68 concentration (5–15% *w*/*w*) and atorvastatin calcium concentration (5–10% *w*/*w*) on phase transition temperature and phase transition interval. Molecular interactions were assessed by Fourier-transform infrared spectroscopy. The in vitro dissolution of the optimized thermoresponsive solid dispersion was assessed using a USP Apparatus II dissolution test. **Results**: Propylene glycol was identified as the optimal thermo-modulating agent, forming a rigid carrier through hydrogen bonding with Pluronic F-68. The optimized thermoresponsive solid dispersion consisted of 10.07% *w*/*w* Pluronic F-68 and 9.98% *w*/*w* atorvastatin calcium. It converted to a solution state at 32 °C. At physiological temperature, the phase transition interval was 111.66 s. Dissolution studies demonstrated that the thermoresponsive solid dispersion enhanced the dissolution profile of atorvastatin calcium within 5 min, 96.6 ± 1.2% compared to 13.8 ± 4.2% for the raw drug. A comparison of process characteristics indicated fewer unit operations and no organic-solvent requirement relative to conventional techniques. **Conclusions**: This approach enhances dissolution performance and eliminates the need for organic solvents through simple manufacturing processes.

## 1. Introduction

The unsatisfactory bioavailability of poorly water-soluble drugs is characteristically ascribed to their diminished solubility within the gastrointestinal tract [1,2]. This negatively affects their utilization during the development of a pharmaceutical dosage form [3]. To tackle this limitation, various pharmaceutical approaches have been invented [4,5]. Solid dispersion is a technique in which the drug is dispersed within an inert carrier matrix in the solid state [6]. It has received considerable attention due to its reported capability to augment the dissolution and bioavailability of lipophilic drugs [6,7,8]. 

Spray drying and hot-melt extrusion are well-established technologies that are applied successfully at commercial scale and account for the majority of the amorphous solid dispersion products currently approved [9]. Nevertheless, these processes entail the use of organic solvents or the application of thermal and mechanical energy. Moreover, in certain cases they could require additional post-processing of the resulting solid mass. Such considerations have driven a continued interest in solvent-free preparation strategies with simple production methods for enhancing the dissolution of poorly water-soluble drugs. Therefore, this study was designed to develop alternative methods to overcome the challenges associated with traditional solid dispersion preparation.

Pluronic (Poloxamer) is a triblock copolymer (PEO–PPO–PEO) that is well known for its thermoresponsive behavior in aqueous media. In water, these copolymers exist as a low-viscosity solution at low temperatures. However, they undergo a sol-to-gel transition upon warming, owing to dehydration of the central PPO blocks, micellization, and subsequent packing [10,11]. Conversely, when poloxamers are dissolved in a nonaqueous solvent, the direction of transition is inverted. The nonaqueous Pluronic solution is rigid at low temperatures and becomes liquid upon heating. This transition was reported by Fahad et al. for Pluronic F-127 dissolved in ethylene glycol. The transition was attributed mainly to hydrogen bonding rather than to the micellar mechanism, as in an aqueous system [12]. The thermoresponsive solid dispersion is designed to be in a solid state after preparation and liquefies upon exposure to physiological body temperature. 

The systematic optimization of thermoresponsive solid dispersions combining Pluronic F-68 with appropriate cosolvents to enhance drug dissolution remains to be explored. In this context, a novel thermoresponsive solid dispersion comprising a polymer (Pluronic F-68) and a thermo-modulating agent (propylene glycol) was developed to address the drawbacks of existing methods. The proposed thermoresponsive solid dispersion is rigid and becomes a solution upon exposure to physiological body temperature. 

Atorvastatin calcium was used as a model drug in the present study due to its reported low aqueous solubility [13,14,15]. It is used in clinical settings to treat hypercholesterolemia and prevent the exacerbation of cardiovascular diseases [16,17]. It exerts its action by inhibiting the HMG-CoA reductase enzyme, thus lowering the de novo synthesis of cholesterol [18]. The reported low drug bioavailability (~14%) necessitates the utilization of a formulation approach to enhance its dissolution [19,20]. 

This work aims to optimize a thermoresponsive solid dispersion containing atorvastatin calcium to examine its potential to enhance the dissolution profile of the poorly water-soluble drug. Stat-Ease 360 software was used to map how the concentrations of Pluronic F-68 and atorvastatin calcium govern the two responses of interest, phase transition temperature and phase transition interval. The formulation identified at the optimum was then prepared and subjected to in vitro dissolution testing, and its manufacturing feasibility was finally weighed against that of the conventional routes described in the literature.

## 2. Materials and Methods

### 2.1. Materials

Atorvastatin calcium (drug) and propylene glycol were supplied by SPIMACO (Qassim, Saudi Arabia) and Winlab Laboratory (Leicestershire, UK), respectively. Ammonium formate and formic acid (buffer for mobile phase) were obtained from Sigma-Aldrich (St. Louis, MO, USA). Acetonitrile (organic mobile phase) was purchased from AppliChem Panreac (Darmstadt, Germany).

### 2.2. Quantification Method for Atorvastatin Calcium

Atorvastatin calcium was assayed on a Dionex™ UHPLC system (Thermo Fisher Scientific, Bedford, MA, USA). Chromatographic separation was achieved on a BEH C18 column (2.1 × 50 mm, 1.7 µm) using a ternary mobile phase of 0.1% formic acid solution, 10 mM ammonium formate buffer, and acetonitrile (10:45:45 *v*/*v*/*v*) eluted at 0.4 mL/min. The column was thermostatted at 30 ± 0.1 °C in a Dionex oven chamber. Absorbance was monitored at 245 nm on a Dionex photodiode array detector. Quantification relied on a linear calibration curve covering 5.0–50.0 µg/mL, for which the coefficient of determination exceeded 0.999.

### 2.3. Identification of Optimum Thermo-Modulating Solvent

Hydroxyl-containing nonaqueous solvents (methanol, ethanol, propanol, and propylene glycol) were selected for screening based on previous studies. These studies demonstrated that poloxamers dissolved in nonaqueous solvents solidify upon cooling and liquefy upon heating. Therefore, this experiment was performed to investigate their potential usage as thermo-modulating agents. Briefly, 100 mg of Pluronic F-68 and 1900 mg of thermo-modulating solvent were placed in a cylindrical glass tube to examine solubilization behavior. The mixture was placed in an incubator set at 40 °C to solubilize the Pluronic F-68 polymer. The solubilized mixtures were placed in a refrigerator set at 8 °C to study their behavior upon cooling. This process was repeated in triplicate, and photographs of the prepared mixture were taken. 

### 2.4. Preparation of Thermoresponsive Solid Dispersion

The predetermined amounts of Pluronic F-68 and propylene glycol, based on the formulation composition, were mixed and maintained at 40 °C until the polymer was fully dissolved. Mild manual agitation was then applied to obtain a homogeneous system, which was determined visually by the formation of a clear solution. Atorvastatin calcium was subsequently incorporated at the same temperature. The resulting liquid formulation was filled into hard gelatin capsules in the liquid state and cooled to allow solidification of the carrier within the capsule. A ternary physical mixture was prepared by gently blending atorvastatin calcium, Pluronic F-68, and propylene glycol at the same ratio as the optimized formulation.

### 2.5. Experimental Design and Optimization

Stat-Ease 360 software (version 25.0.7; Stat-Ease Inc., Minneapolis, MN, USA) was used to investigate the impact of independent factors on the measured responses of thermoresponsive solid dispersion. A central composite design was used to cover the selected range with fewer experimental runs while retaining adequate predictive capability. Two independent variables were studied: Pluronic F-68 concentration (5–15% *w*/*w*) and atorvastatin calcium concentration (5–10% *w*/*w*). Two responses were evaluated: phase transition temperature (°C) and phase transition interval (seconds). The suggested thirteen formulations are presented in Table 1. The model was chosen based on the statistical analysis conducted using Stat-Ease 360 software, which included analysis of variance (ANOVA), comparison of predicted versus adjusted R^2^ values, lack-of-fit tests, and an adequate precision (signal-to-noise ratio > 4, as desired). The optimized formulation was selected using Stat-Ease 360 software based on the following criteria: maximum phase transition temperature and atorvastatin calcium concentration while minimizing phase transition interval. 

### 2.6. Measuring Phase Transition Temperature

Prior to measurement, each dispersion was transferred to a test tube and refrigerated until a rigid system had formed. At the beginning of the experiment, the temperature was controlled using a water bath set to 28 ± 0.1 °C, and the test tubes were held in place on a rack in the bath. The thermoresponsive solid dispersions were left for at least 5 min for temperature equilibration. The test tubes were visually checked to verify whether conversion from the rigid to the solution state had occurred. The bath was subsequently warmed in increments of 0.5 ± 0.1 °C and the inspection repeated after every increment. The temperature at which conversion occurred was recorded as the phase transition temperature of that thermoresponsive solid dispersion system.

### 2.7. Measuring Phase Transition Interval

The phase transition interval was determined separately for every dispersion with the water bath thermostatted at 37 ± 0.1 °C. This condition simulates the thermal conditions encountered in vivo. A tube containing the dispersion was immersed in the bath, and the time elapsed until complete conversion of the rigid mass to the solution state was recorded.

### 2.8. Thermoresponsive Transition Assessment 

The test was performed on the optimized thermoresponsive solid dispersion to investigate transition behavior after oral administration under physiological conditions. Phosphate buffer (pH 6.8) was preheated to 37 ± 0.5 °C, and 100 mL was transferred to a 150 mL beaker. The optimized thermoresponsive solid dispersion was filled into a hard gelatin capsule and wrapped with a wire sinker. After that, it was placed in a glass beaker, and photographs were taken to document transition behaviors.

### 2.9. FTIR Spectroscopy

The molecular interactions governing the solid-to-liquid transition were probed by Fourier-transform infrared (FTIR) spectroscopy. Atorvastatin calcium, Pluronic F-68, propylene glycol, the ternary physical mixture, and the drug-free and drug-loaded thermoresponsive solid dispersions in each of their two physical states were analyzed. Spectra were acquired on a PerkinElmer Spectrum-100 spectrometer (Waltham, MA, USA) and plotted in Excel across the 750–4000 cm^−1^ range.

### 2.10. In Vitro Dissolution

A USP Apparatus II dissolution tester (LOGAN Inst. Corp., Somerset, NJ, USA) was used to study the dissolution profile of atorvastatin calcium from the raw drug and the optimized thermoresponsive solid dispersion. The test agents containing 40 mg of the drug were placed in a hard gelatin capsule and then surrounded with a sinker to prevent the capsule from floating. A dissolution study was performed using preheated phosphate buffer (pH 6.8, 900 mL) as the dissolution medium at 37 ± 0.5 °C. The paddle speed was set to 50 rpm. Samples were withdrawn from the dissolution medium using a syringe equipped with a 10 µm filter at predetermined intervals (5, 10, 15, 30, 45, and 60 min). Drug concentration was estimated using the UHPLC method. 

### 2.11. Use of Generative AI

Claude (Anthropic) was used during preparation of this manuscript to assist in identifying relevant literature and for language editing. All cited references and all statements were checked by the authors against the primary sources. The output was reviewed and edited, and the authors take full responsibility for its content.

## 3. Results and Discussion

### 3.1. Identification of Optimum Thermo-Modulating Solvent

**Figure 1:**
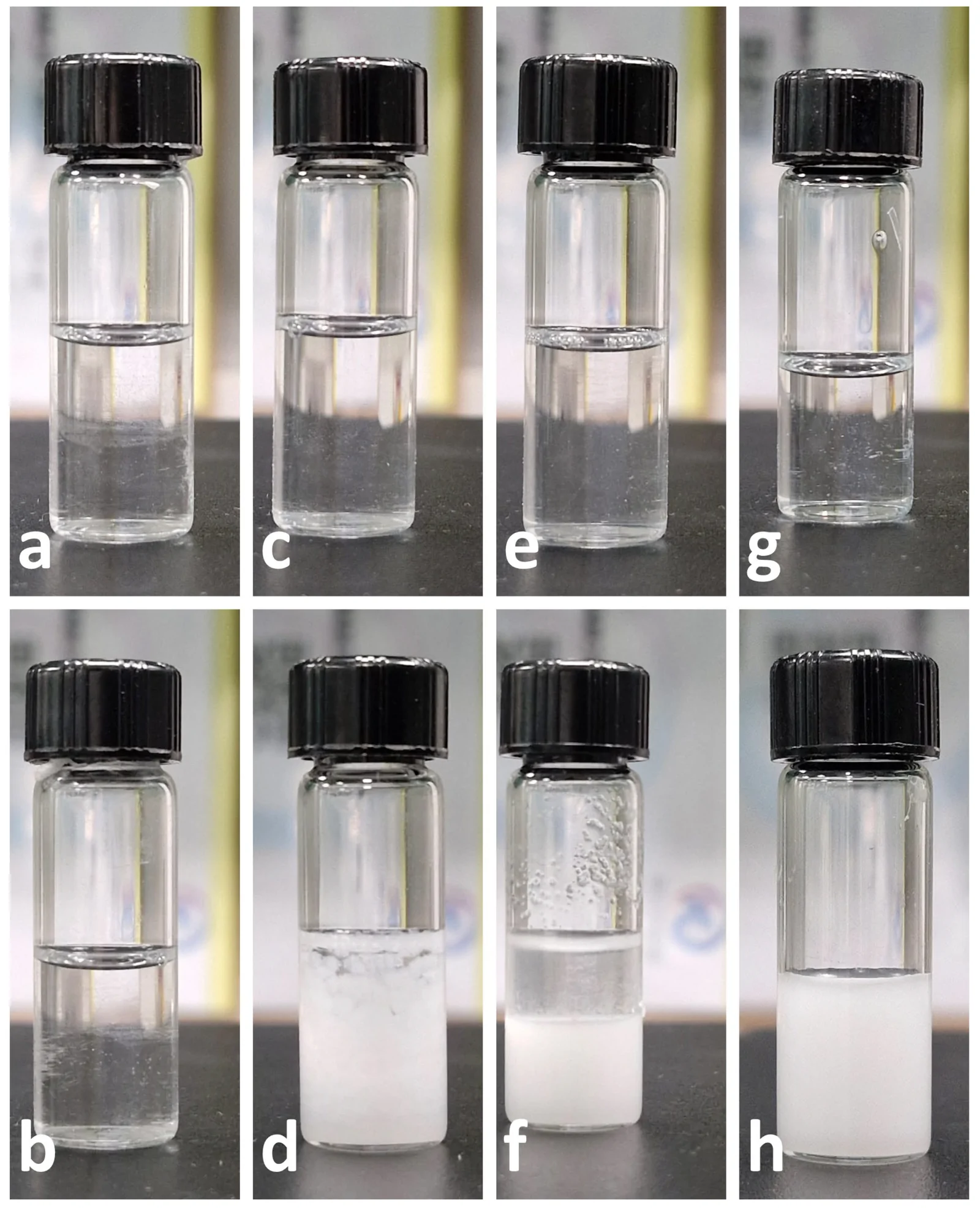
Visual appearance of Pluronic F-68 mixtures with different thermo-modulating solvents (**a**,**b**) methanol, (**c**,**d**) ethanol, (**e**,**f**) propanol, and (**g**,**h**) propylene glycol after heating (**top row**) and cooling (**bottom row**).

The solubilization behavior of Pluronic F-68 in various hydroxyl-containing nonaqueous solvents (methanol, ethanol, propanol, and propylene glycol) was assessed to select the optimal nonaqueous solvent for achieving the desired thermoresponsive performance. Figure 1 presents the visual appearance of Pluronic F-68 mixtures with different hydroxyl-containing nonaqueous solvents during heating and cooling cycles. The Pluronic F-68 mixtures formed a clear solution upon incubation at an elevated temperature, as shown in the top row of Figure 1. This indicates a complete solubilization of Pluronic F-68 in hydroxyl-containing nonaqueous solvents and the formation of favorable interactions [21,22]. 

**Figure 2:**
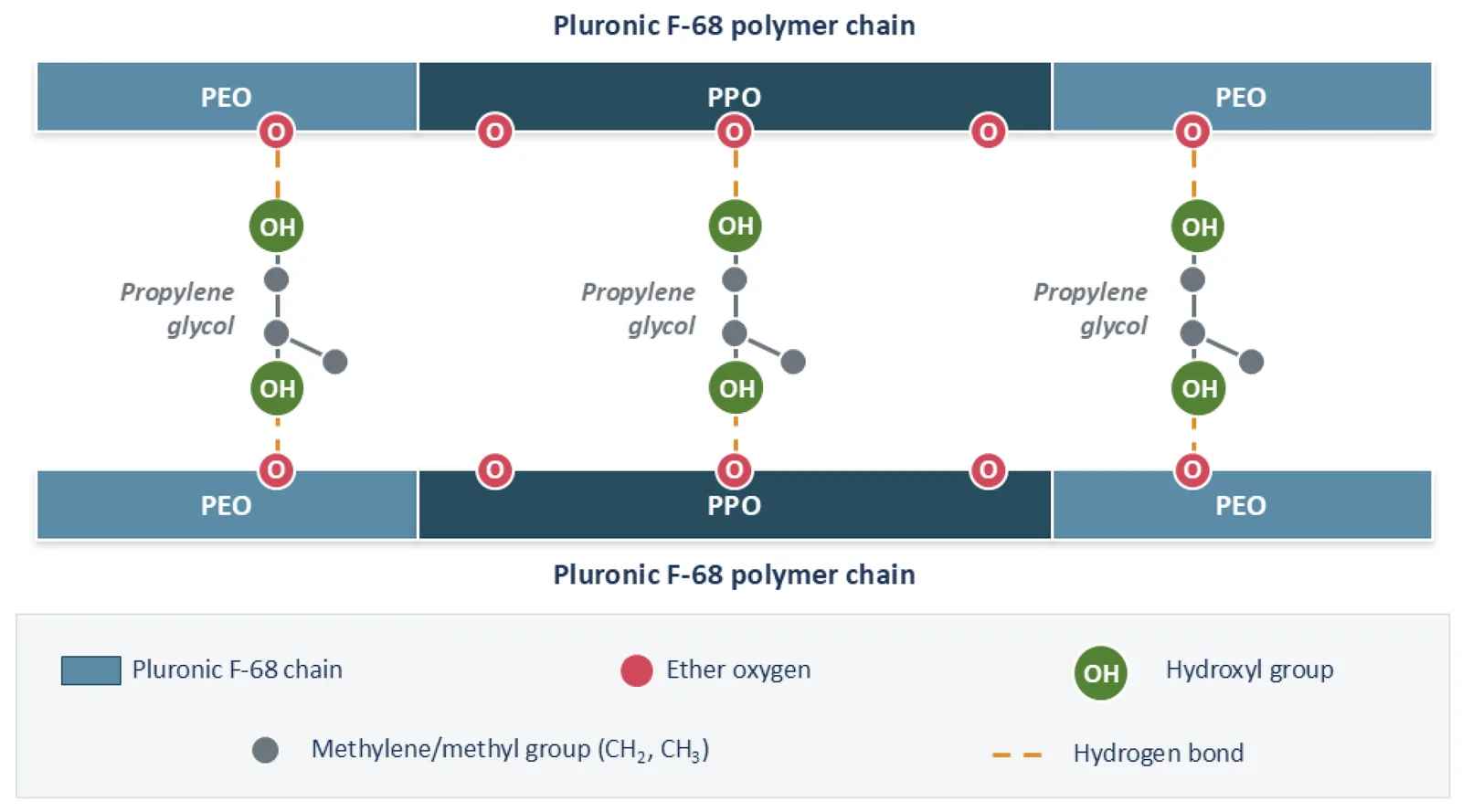
Mechanism of diol-mediated hydrogen-bonding network formation within thermoresponsive solid dispersion system.

The physical appearance of the prepared mixtures changed significantly upon cooling. Figure 1b shows that the Pluronic F-68/methanol system remained in a solution state with no evidence of transformation. However, Figure 1d,f demonstrate that the Pluronic F-68/ethanol and Pluronic F-68/propanol mixtures failed to form a homogeneous, rigid carrier system, with polymer chains precipitating on the wall and at the bottom of the glass container, respectively. In contrast, Figure 1h shows that the Pluronic F-68/propylene glycol mixture formed a rigid carrier system that resisted flow [23]. The unique rigid carrier system could result from the dual hydroxyl functionality of propylene glycol. This enables the formation of intermolecular hydrogen-bonding networks involving the two hydroxyl groups of propylene glycol and the ether oxygens of Pluronic F-68 (Figure 2). Therefore, propylene glycol was selected as a thermo-modulating agent to prepare a rigid carrier system.

### 3.2. Models’ Selection for the Studied Responses

**Figure 3:**
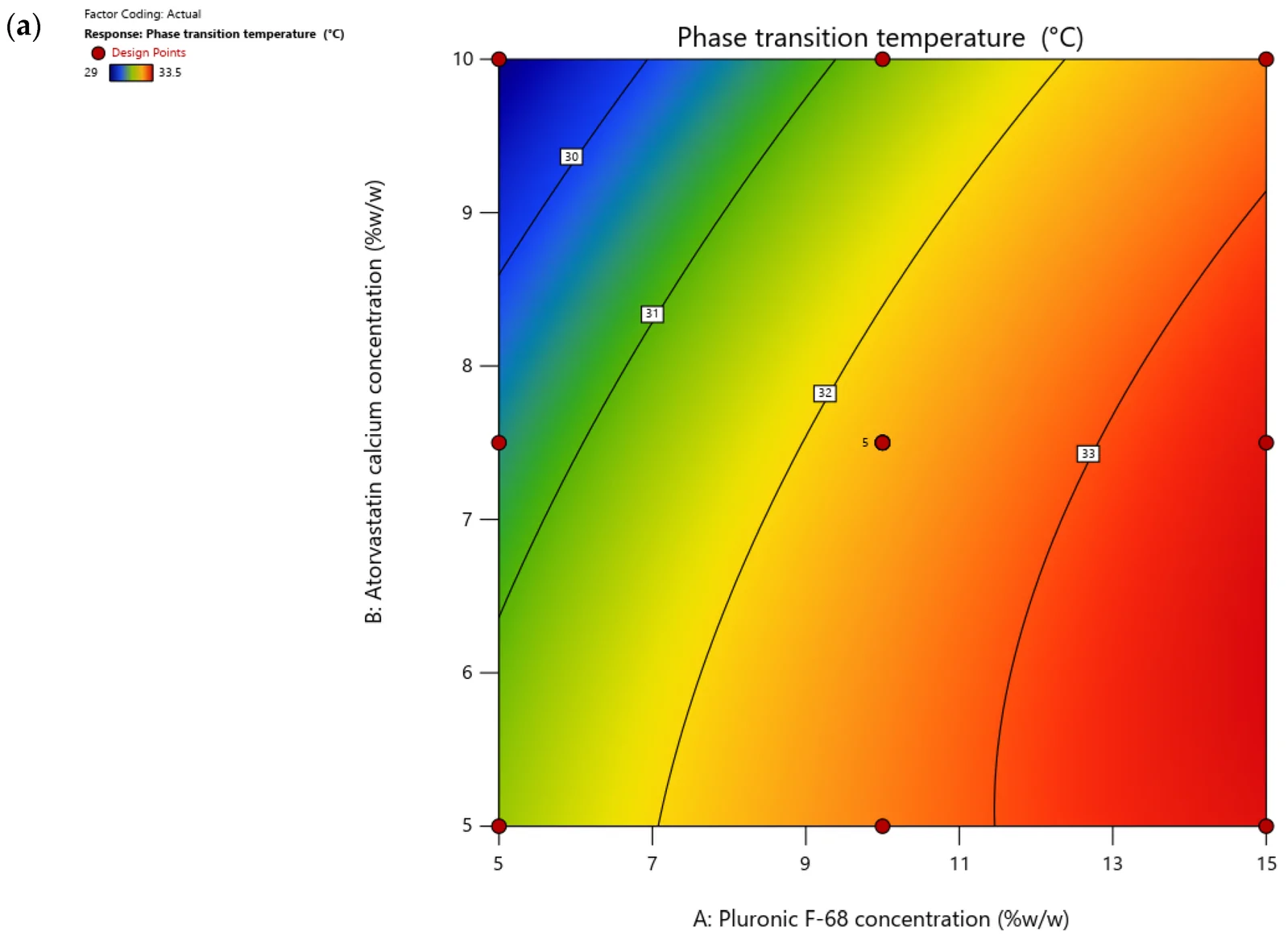

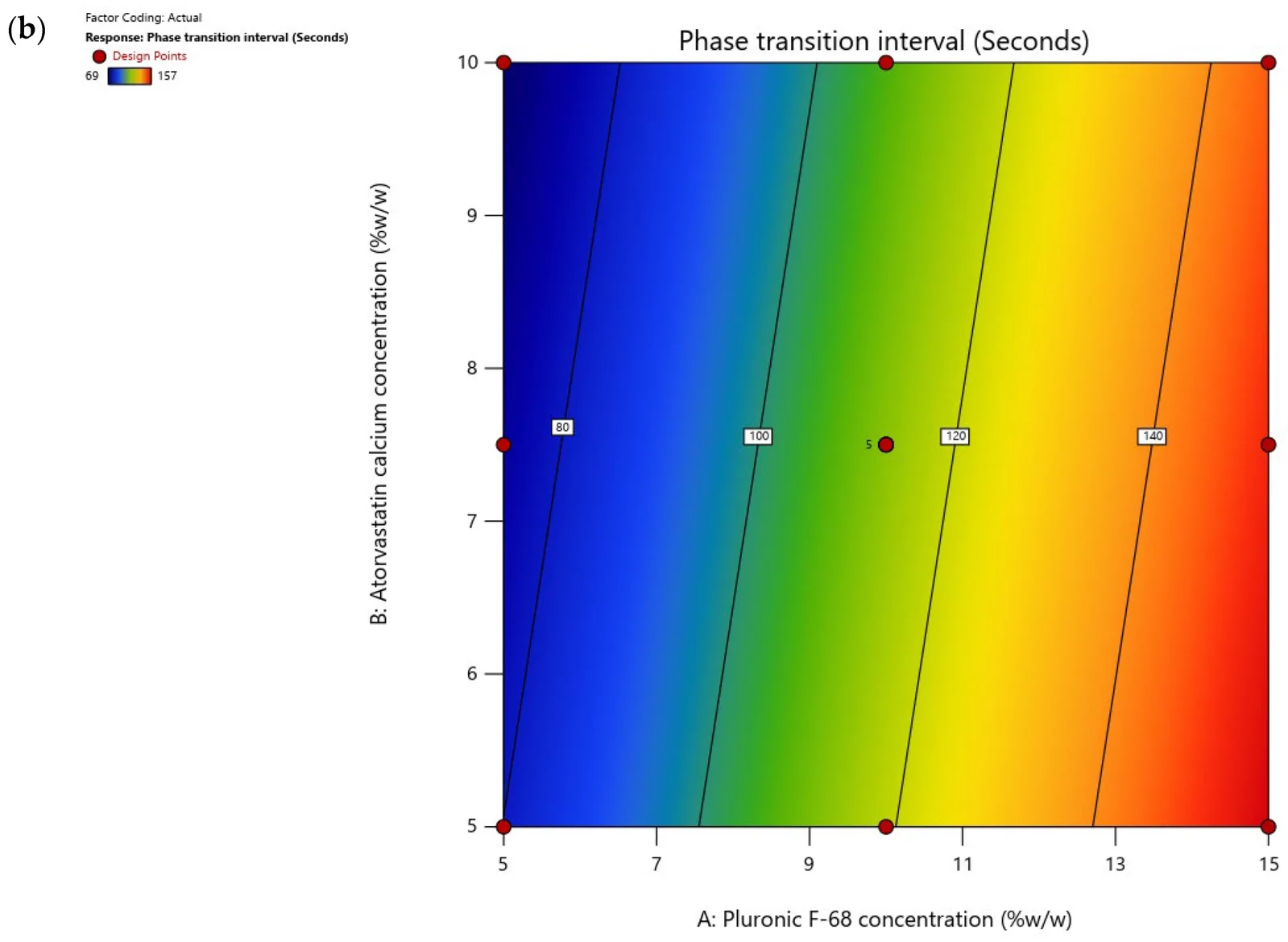
Contour plots of (**a**) phase transition temperature and (**b**) phase transition interval.

The suggested thermoresponsive solid dispersions (13 runs) were prepared, and the phase transition temperature and interval are presented in Table 2. The suitability of the polynomial models was evaluated using the sequential model sum of squares, the lack-of-fit test, and the model summary statistics. For the phase transition temperature, the quadratic model was selected as the highest-order model that was both significant and free of aliasing, exhibiting a strong correlation (R^2^ = 0.9707), close agreement between the adjusted (0.9498) and predicted (0.8523) R^2^ values, an adequate precision of 23.58, and a non-significant lack of fit (*p* = 0.4990). In contrast, for the phase transition interval, the linear model was adopted, providing a high predicted R^2^ (0.9311), close agreement with the adjusted R^2^ (0.9506), an adequate precision of 29.59, and a non-significant lack of fit (*p* = 0.7449). Table 3 summarizes the statistical model selected for each response together with the corresponding fit statistics. Moreover, Figure 3 shows the contour plots of the phase transition temperature and the phase transition interval.

### 3.3. Phase Transition Temperature

**Figure 4:**
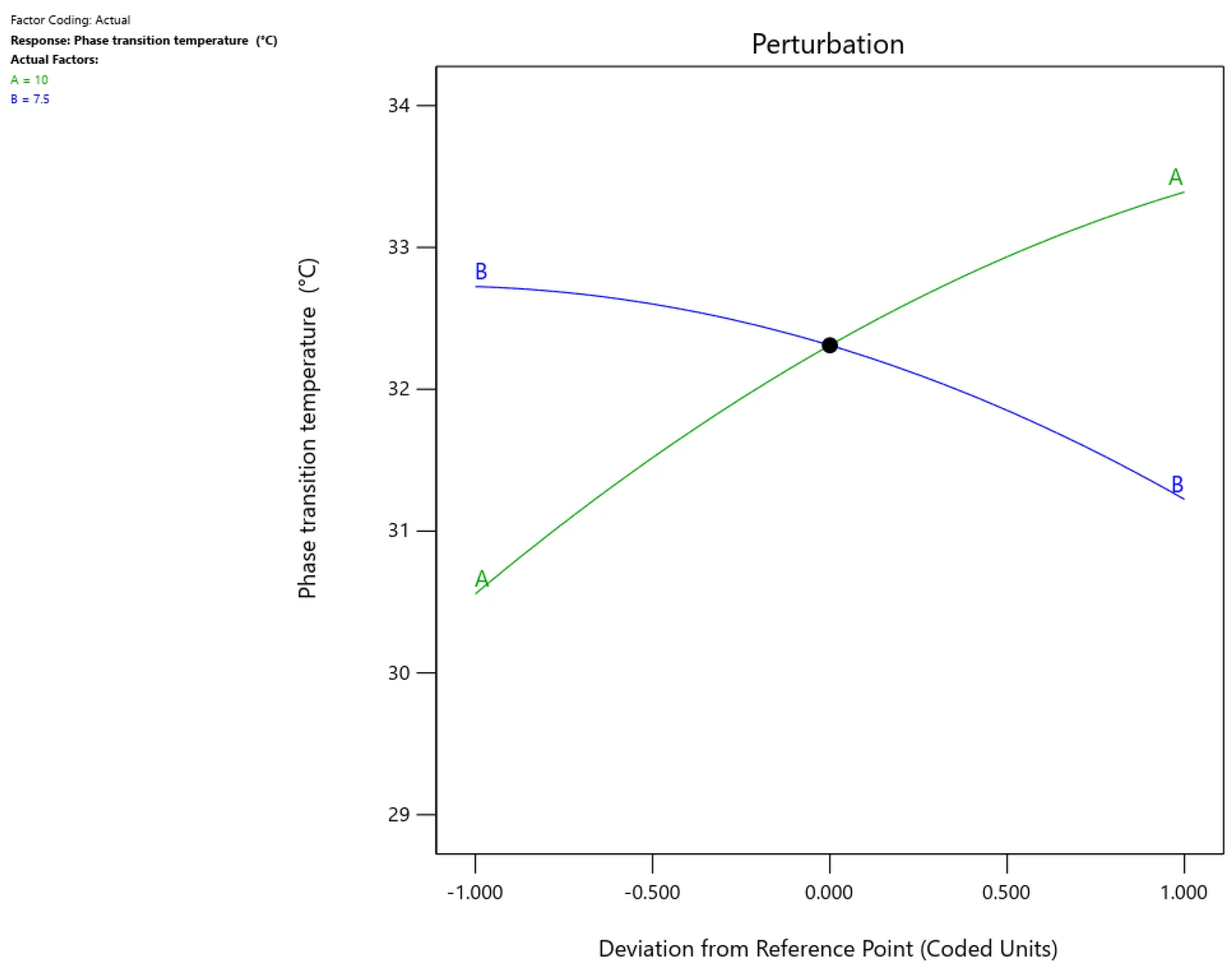
Perturbation plots illustrate the impact of Pluronic F-68 concentration (curve A) and atorvastatin calcium concentration (curve B) on phase transition temperature (°C).

Phase transition temperatures recorded across the prepared thermoresponsive solid dispersions spanned from 29 to 33.5 °C. Raising the Pluronic F-68 content shifted this response significantly upward, whereas raising the atorvastatin calcium content shifted it significantly downward (Table 4). This aligns with the perturbation plots (Figure 4), which demonstrate a positive slope for Pluronic F-68 concentration and a negative slope for atorvastatin calcium concentration. Furthermore, the coded factors equation quantitatively confirms the greater impact of Pluronic F-68 concentration on phase transition temperature, as indicated by a larger coefficient magnitude (+1.42) than that of atorvastatin calcium concentration (−0.75) (Equation (1)).

Phase transition temperature = 32.31 + 1.42 × Pluronic F-68 concentration − 0.75 × Atorvastatin calcium concentration + 0.38 × Pluronic F-68 concentration × Atorvastatin calcium concentration − 0.34 × Pluronic F-68 concentration^2^ − 0.34 × Atorvastatin calcium concentration^2^ (1)

The coded regression equation for the phase transition temperature (Equation (1)) provides mechanistic insight into the influence of the formulation variables. The positive coefficient of Pluronic F-68 (+1.42, *p* < 0.0001) is the dominant term in the equation. The observed increase in the phase transition temperature with increasing polymer concentration could be ascribed to the greater number of Pluronic F-68 chains in proximity within the thermoresponsive solid dispersion. This promotes a more extensive intermolecular hydrogen-bonding network between the ether oxygens of Pluronic F-68 and the hydroxyl groups of propylene glycol. Therefore, a solid dispersion system forms a rigid matrix that necessitates a higher temperature to break these hydrogen bonds. This is supported by previously reported studies in which increasing the Pluronic F-68 concentration resulted in a more rigid carrier system than at lower concentrations [24,25]. On the other hand, the negative coefficient of atorvastatin calcium (−0.75, *p* = 0.0003) indicates that increasing its concentration could disrupt the polymer’s organization. This indicates an antagonizing effect of atorvastatin calcium on the rigidity of the carrier system. Therefore, a lower temperature is required to trigger the transition to a solution state at higher drug concentrations than at lower concentrations. This agrees with previous studies reporting that incorporating drugs into the polymer matrix reduces its integrity [26]. The significant positive interaction term (+0.38, *p* = 0.0276) indicates that both components contribute to the same hydrogen-bonding network. Therefore, the effect of each component on the phase transition temperature depends on the concentration of the other. The second-order terms of both factors were small, equal, and negative (−0.34) with no statistically significant effect (*p* = 0.0777). This indicates that they had only a low impact on the phase transition temperature rather than a strong quadratic dependence on concentration.

### 3.4. Phase Transition Interval

**Figure 5:**
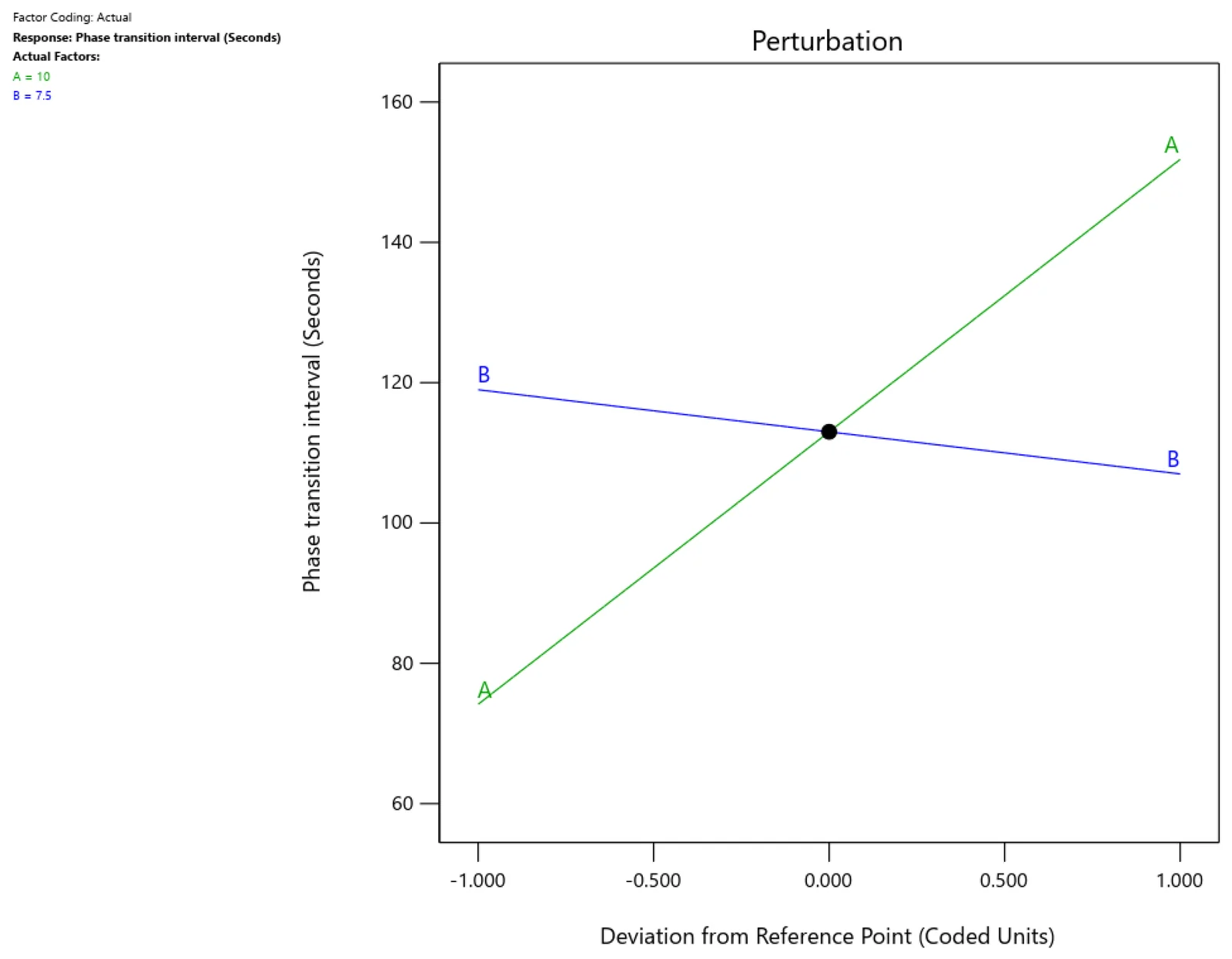
Perturbation plots illustrate the impact of Pluronic F-68 concentration (curve A) and atorvastatin calcium concentration (curve B) on phase transition interval (seconds).

The measured phase transition interval for the prepared rigid carrier systems ranged from 69 to 157 s. Statistical analysis revealed that increasing the concentration of Pluronic F-68 significantly increased the phase transition interval of the prepared rigid carrier system (Table 4). On the other hand, increasing the concentration of atorvastatin calcium significantly reduced the phase transition interval of the prepared rigid carrier system (Table 4). This is consistent with the perturbation plots (Figure 5), which demonstrate a positive slope for Pluronic F-68 concentration and a negative slope for atorvastatin calcium concentration. Furthermore, the coded factors equation quantitatively confirms the predominant impact of Pluronic F-68 concentration on phase transition interval, indicated by a larger coefficient magnitude (+38.83) compared with that of atorvastatin calcium concentration (−6.00) (Equation (2)).

Phase transition interval = 113.00 + 38.83 × Pluronic F-68 concentration − 6.00 × Atorvastatin calcium concentration(2)

The phase transition interval was measured to ensure rapid conversion of the rigid carrier system to a solution state upon exposure to body temperature (37 °C). The positive coefficient of Pluronic F-68 (+38.83, *p* < 0.0001) is the dominant term in the equation. The observed increase in the phase transition interval with increasing polymer concentration could be ascribed to the intermolecular hydrogen-bonding network that imparts rigidity to the carrier system. Owing to the long chain length of Pluronic F-68, increasing its concentration strengthens the entangled hydrogen-bonding network and increases the strength of the rigid carrier system. Therefore, a longer transition time is required for conversion to the liquid state. On the other hand, the negative coefficient of atorvastatin calcium (−6.00, *p* = 0.0420) indicates that increasing its concentration could disrupt the arrangement of the carrier system through its competition with propylene glycol and Pluronic F-68 for hydrogen bonding. This reduces the matrix strength of the carrier system. Therefore, less time is required to disrupt the hydrogen-bonding network, and the phase transition interval is correspondingly shorter. This agrees with a previously reported study, in which incorporating the drug into the polymer matrix weakened the network structure and reduced the intermolecular forces between polymer chains [27]. 

### 3.5. Selection of an Optimized Thermoresponsive Solid Dispersion

**Figure 6:**
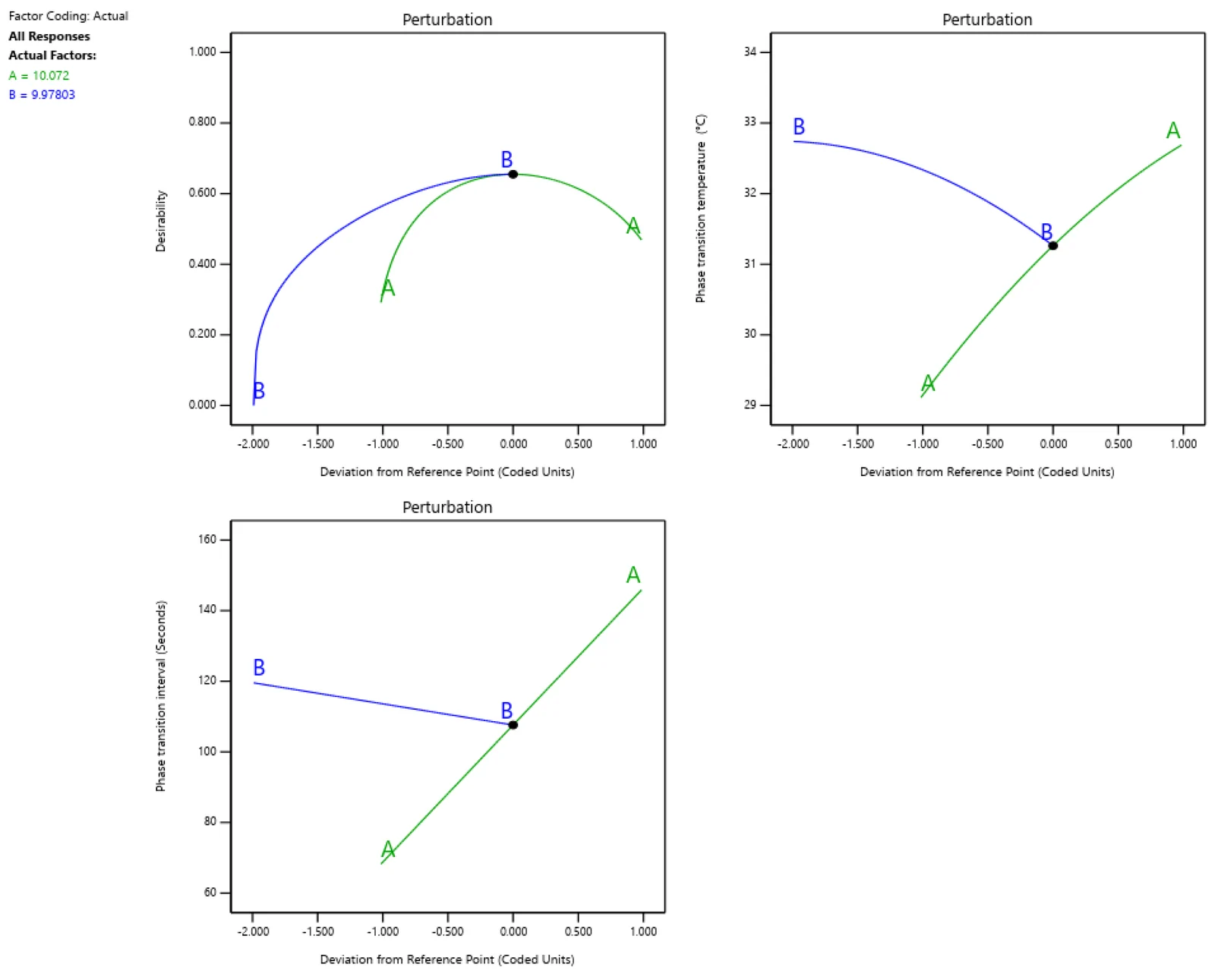
Perturbation plots showing the effect of Pluronic F-68 concentration (curve A) and atorvastatin calcium concentration (curve B) on desirability, phase transition temperature, and phase transition interval for the optimized thermoresponsive solid dispersion.

Stat-Ease 360 software was used to perform numerical optimization and suggest an optimized formulation based on the following criteria. The maximum atorvastatin calcium concentration was chosen to attain higher drug loading and reduce the number of units per dose. In addition, maximizing the phase transition temperature was preferred to avoid premature conversion of the formulation to the solution state during storage. Finally, a minimum phase transition interval is needed to ensure rapid formulation conversion to a solution state once exposed to body temperature in vivo. Figure 6 shows the perturbation plots for desirability, phase transition temperature, and phase transition interval at the optimized point where Pluronic F-68 and atorvastatin calcium concentrations meet the selected criteria. The highest desirability value (0.655) was attained at a Pluronic F-68 concentration of 10.07% *w*/*w* and an atorvastatin calcium concentration of 9.98% *w*/*w*. The optimized thermoresponsive solid dispersion is expected to achieve a phase transition temperature of 31.26 °C and convert to a solution state within 107.58 s when exposed to physiological temperature (37 °C). 

### 3.6. Validation of Optimized Formulation

The actual values of the phase transition temperature and phase transition interval were determined for a freshly prepared optimized thermoresponsive solid dispersion. The values of actual and predicted means for predetermined responses are presented in Table 5. The closeness values for both means indicate that the suggested models for the measured responses were accurate across the design space for each response. Since the formulation exhibits a phase transition temperature of 32.00 °C, it should be stored and transported below this temperature under controlled cool conditions to preserve its solid state. Therefore, refrigerated storage (2–8 °C) provides a wide thermal margin, and the required storage and transportation conditions would be specified on the product packaging. 

### 3.7. Mechanism of Thermoresponsive Performance

**Figure 7:**
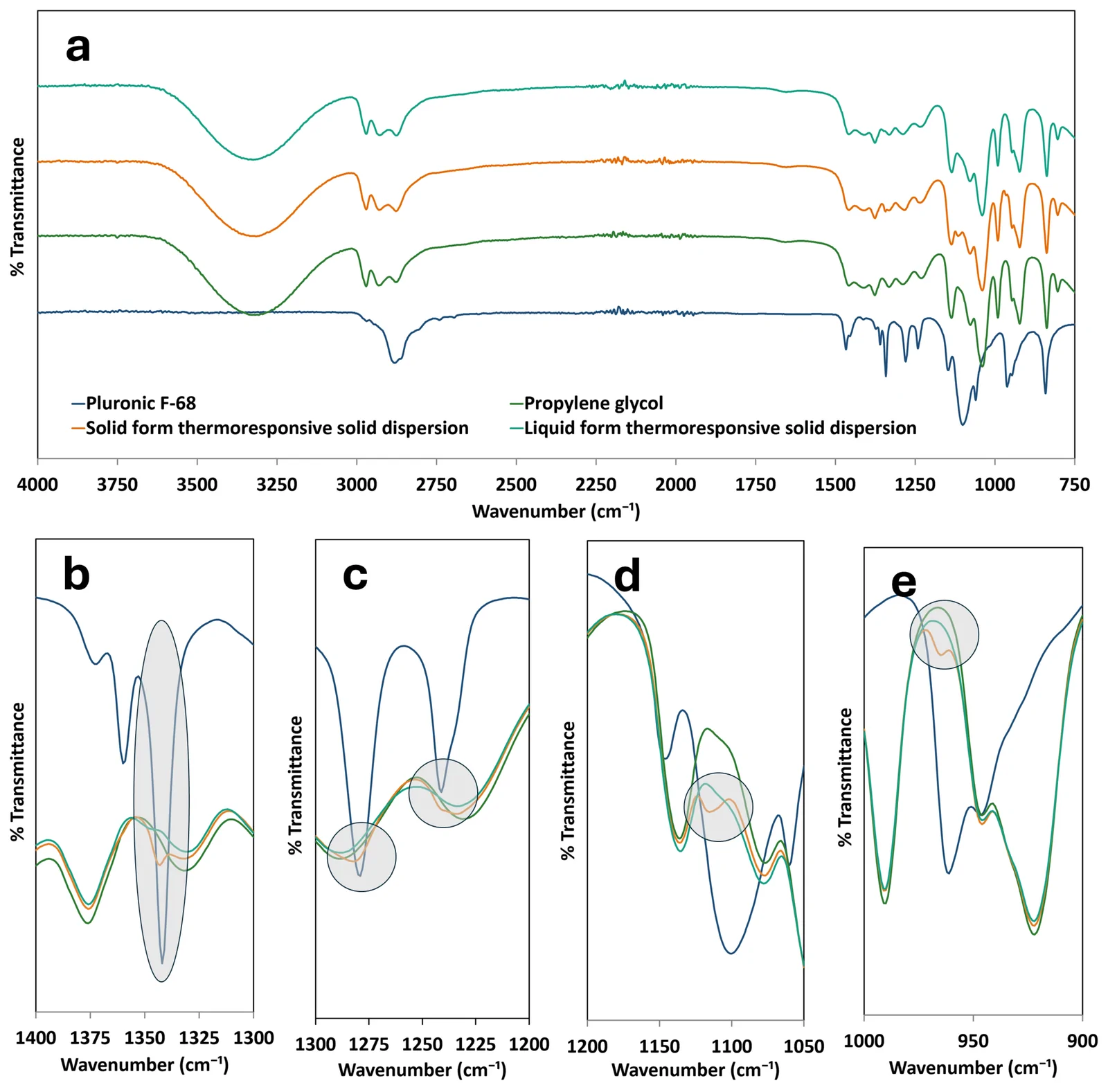
FTIR spectra of pure Pluronic F-68, propylene glycol, and the thermoresponsive solid dispersion in its solid and liquid states. (**a**) Full range (4000–750 cm^−1^). (**b**–**e**) Expanded diagnostic crystalline-PEO regions: CH_2_ wagging (1400–1300 cm^−1^); CH_2_ twisting/1242 cm^−1^ shoulder (1300–1200 cm^−1^); C–O–C stretching (1200–1050 cm^−1^); CH_2_ rocking (1000–900 cm^−1^), respectively.

FTIR spectroscopy was used as the principal structural probe for the trigger mechanism of the Pluronic F-68/propylene glycol carrier. This could provide a clear understanding of the mechanism of the thermoresponsive solid dispersion’s solid-to-liquid transition. The FTIR spectrum of the drug-free formulation was obtained to eliminate the potential impact of the drug on the carrier system’s behavior. Moreover, FTIR spectra of Pluronic F-68 and propylene glycol were obtained to identify characteristic peaks and determine the non-interfering regions for mechanistic insight. In addition, the characteristic peaks of Pluronic F-68 in the optimized thermoresponsive solid dispersion were studied in this prepared drug-free system. The Pluronic F-68 was solubilized in propylene glycol at a ratio similar to the optimum formulation, and FTIR spectra of the solid and liquid forms of the formulation were obtained. Figure 7 shows the FTIR spectra of Pluronic F-68, propylene glycol, and the thermoresponsive solid dispersion in both physical states. Pluronic F-68 is a triblock PEO–PPO–PEO copolymer with hydrophilic PEO blocks that crystallize in the solid state. Its spectrum showed a crystalline C–O–C triplet at 1146, 1100, and 1060 cm^−1^, together with the CH_2_ wagging doublet at 1360 and 1342 cm^−1^, CH_2_ twisting at 1279 and 1242 cm^−1^, and the CH_2_ rocking doublet at 961 and 947 cm^−1^.

The primary crystallinity marker of Pluronic F-68 (CH_2_ wagging doublet at 1342/1360 cm^−1^) was monitored to check the crystalline state of the PEO block portion. It has been reported that merging two peaks into one correlates with decreased crystallinity [28]. Even though propylene glycol has a characteristic peak in this range, the preservation of CH_2_ wagging at 1342 cm^−1^ indicates retention of this crystalline structure at low temperature [28,29]. In addition, the characteristic Pluronic F-68 twisting peak at 1242 cm^−1^ in the solid form of the thermoresponsive solid dispersion provides additional evidence of crystalline PEO conformation [29]. Finally, the presence of the CH_2_ rocking doublet at 961 and 947 cm^−1^ provides an independent third confirmation that the solid carrier is crystalline PEO and that melting destroys this order. In the current study, the doublet and triplet peaks observed in the C–O–C ether stretching region (1050–1200 cm^−1^) correspond to raw propylene glycol and Pluronic F-68, respectively. The solid form showed a C–O–C triplet, whereas the liquid form of thermoresponsive solid dispersion showed only a doublet. This finding provides indirect evidence of hydrogen bonding between the ether oxygens of Pluronic F-68 and the propylene glycol hydroxyl groups in the solid form of thermoresponsive solid dispersion [30,31].

### 3.8. Thermoresponsive Transition Assessment

**Figure 8:**
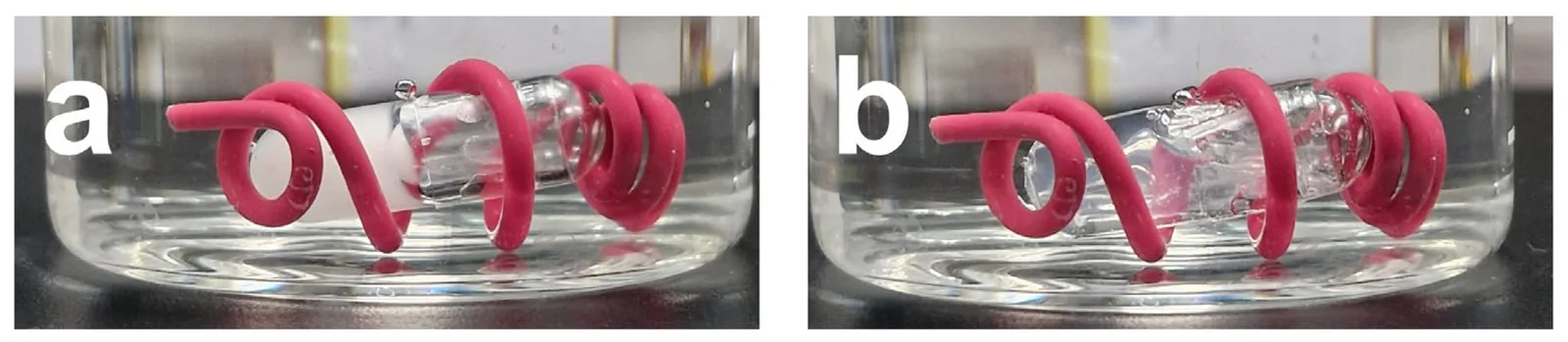
Physical appearance of the prepared formulation (**a**) before and (**b**) after complete liquefaction and release of the contents at physiological body temperature (37 °C).

Figure 8a shows that the thermoresponsive solid dispersion exists in a rigid state at the beginning of the experiment. Rapid conversion to a solution state was observed upon exposure to physiological temperature (37 °C) due to the breakdown of hydrogen bonding between Pluronic F-68 and propylene glycol. The present study indicated that the optimized thermoresponsive solid dispersion can be converted to a solution state under simulated physiological conditions. Figure 8b shows that the rigid carrier system had become a solution by the end of the experiment, in less than 5 min. 

### 3.9. In Vitro Dissolution

**Figure 9:**
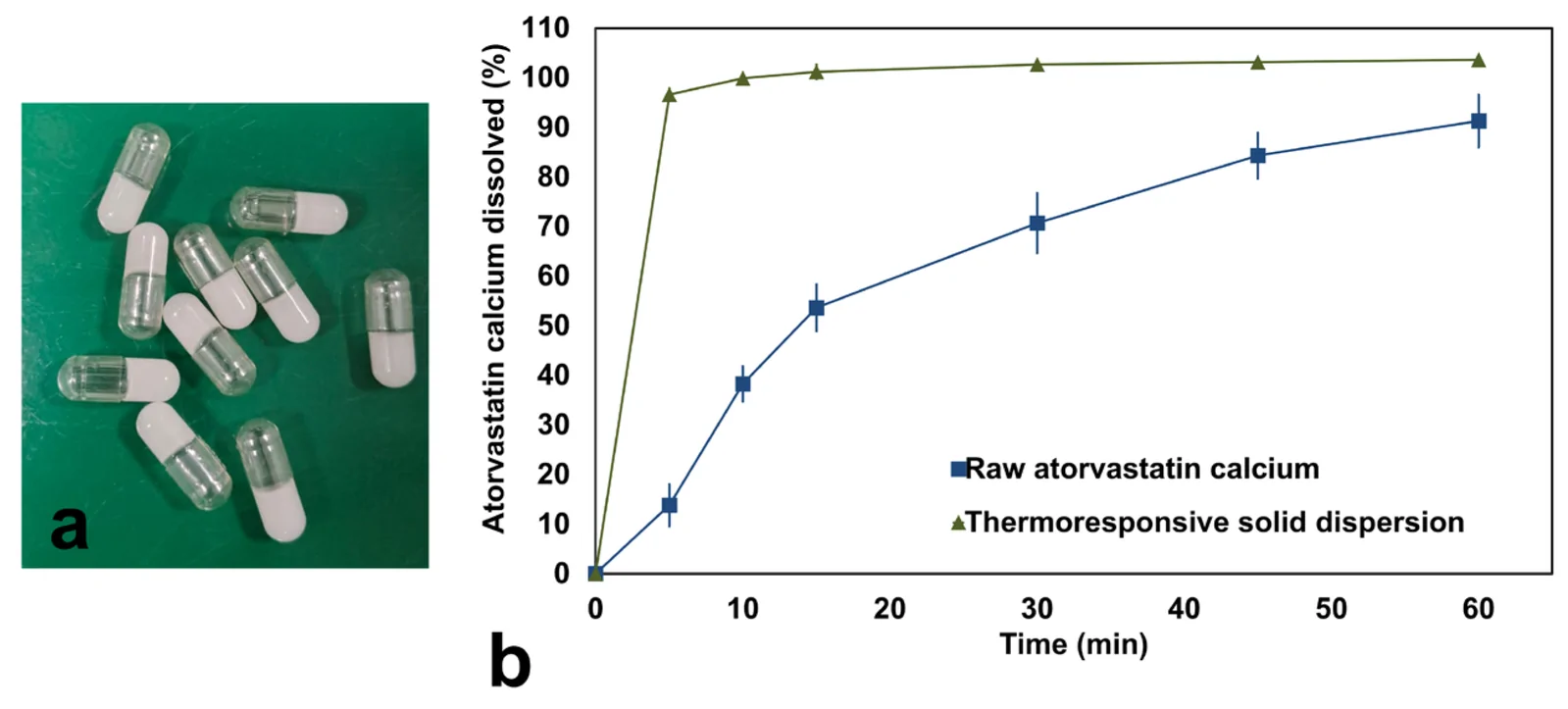
(**a**) Capsules filled with a thermoresponsive solid dispersion. (**b**) In vitro dissolution profile of atorvastatin calcium from a capsule containing the raw atorvastatin calcium or the thermoresponsive solid dispersion. Each data point represents the mean ± standard deviation (n = 3).

Capsules filled with the thermoresponsive solid dispersion are shown in Figure 9a, and the in vitro dissolution profiles of the raw atorvastatin calcium and the thermoresponsive solid dispersion are shown in Figure 9b. Compared with the raw atorvastatin calcium, the optimized thermoresponsive solid dispersion increased the extent of drug dissolution within 5 min from 13.8 ± 4.2% to 96.6 ± 1.2%. This could be attributed to a prompt transition mechanism triggered by physiological thermal conditions (37 °C). The complete conversion of the formulation to a solution, shown in Figure 8, eliminates the mass-transfer limitations of undissolved rigid systems. The drug is delivered within the thermoresponsive solid dispersion, which facilitates dissolution within the dissolution medium. Propylene glycol and Pluronic F-68 are co-released and act as solubilizers in the diffusion layer. Their local concentration remains high before bulk dilution. This could enhance dissolution performance relative to conventional approaches.

### 3.10. Impact of Atorvastatin Calcium on Thermoresponsive Solid Dispersion

**Figure 10:**
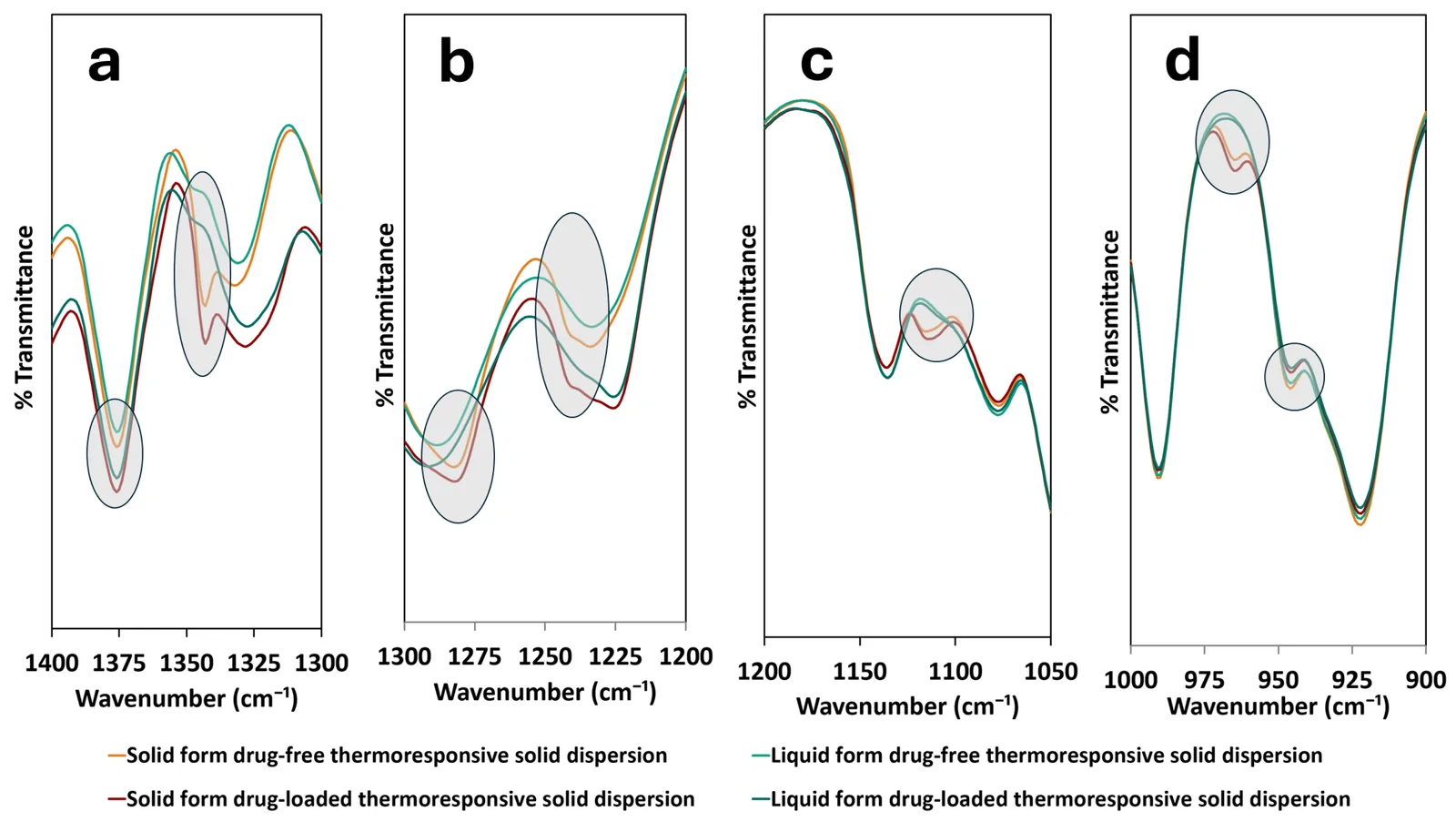
FTIR spectra of drug-free and drug-loaded thermoresponsive solid dispersions in their solid and liquid states. (**a**–**d**) Expanded diagnostic crystalline-PEO regions: CH_2_ wagging (1400–1300 cm^−1^); CH_2_ twisting/1242 cm^−1^ shoulder (1300–1200 cm^−1^); C–O–C stretching (1200–1050 cm^−1^); CH_2_ rocking (1000–900 cm^−1^), respectively.

**Figure 11:**
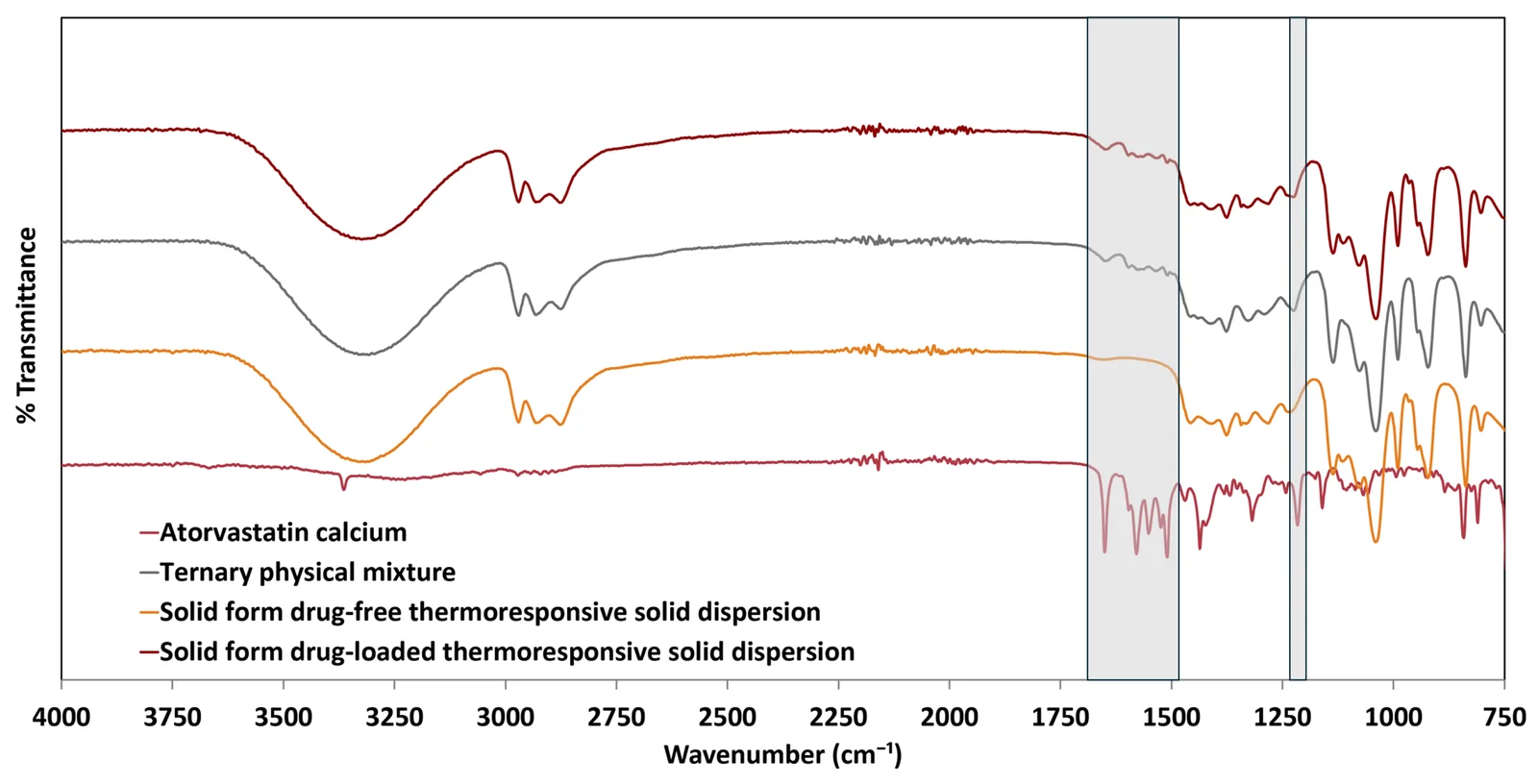
FTIR spectra of pure atorvastatin calcium, ternary physical mixture, the solid form of drug-free thermoresponsive solid dispersion, and the solid form of drug-loaded thermoresponsive solid dispersion.

To confirm that atorvastatin calcium loading into the thermoresponsive solid dispersion does not perturb this mechanism, the four diagnostic regions were compared between the drug-free and drug-loaded carriers in both physical states (Figure 10). The crystalline markers were retained identically in the solid forms and lost identically in the liquid forms in both drug-free and drug-loaded thermoresponsive solid dispersions. This confirms that the solid-to-liquid phase transition is not significantly influenced by the loaded atorvastatin calcium [32,33]. The drug–carrier interaction was then examined by the spectra of pure atorvastatin, the drug-free carrier, the ternary physical mixture, and the thermoresponsive solid dispersion (Figure 11). Two characteristic atorvastatin calcium regions were monitored: the C=O/amide region (1500–1700 cm^−1^) and the C–F band at 1216 cm^−1^ (1200–1300 cm^−1^). The former comprises the amide I C=O band at 1651 cm^−1^, the amide II/aromatic C=C band at 1579 cm^−1^, and the aromatic C=C band at 1510 cm^−1^ [33,34]. The characteristic atorvastatin calcium peaks were preserved at their unchanged positions, with no new bands observed in either the physical mixture or the solid formulation. This finding indicates that atorvastatin calcium is incorporated into the thermoresponsive solid dispersion without chemical interaction [33].

### 3.11. Industrial Sustainability Assessment

**Figure 12:**
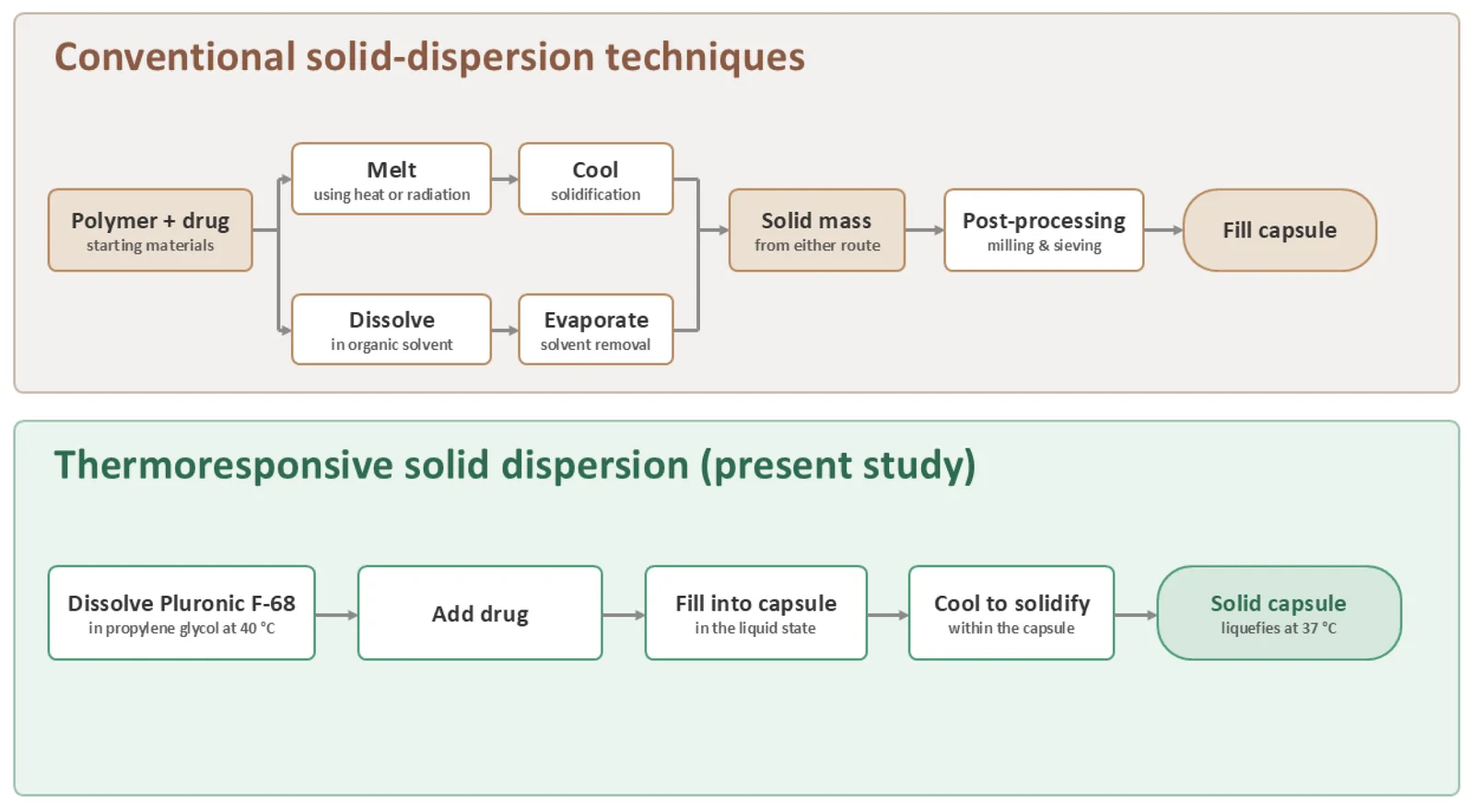
Schematic comparison of the process steps involved in conventional solid-dispersion techniques (**upper panel**) and the thermoresponsive solid dispersion developed in the present study (**lower panel**).

The process characteristics of the thermoresponsive solid dispersion were compared with those of conventional solid-dispersion techniques reported for atorvastatin calcium. For each conventional method, a representative published preparation of atorvastatin calcium is cited, and the comparison is summarized in Table 6 and illustrated schematically in Figure 12. The comparison considers categorical process attributes, namely the requirement for organic solvents, the nature of the thermal or energy input, the number of unit operations, and the requirement for post-processing. As shown in Table 6, conventional techniques such as spray drying and solvent evaporation require organic solvents. On the other hand, hot-melt extrusion, freeze-drying, microwave-induced, and fusion methods are solvent-free approaches. However, all of these routes require active energy input, ranging from heated drying gas and reduced-pressure evaporation to vacuum sublimation, mechanical shear, and dielectric heating. In addition, most of these methods may require downstream milling and sieving to obtain a uniform powder. In contrast, the present formulation is prepared in a single vessel using mild warming at 40 °C. Furthermore, it requires no organic solvents and solidifies without any post-processing step. Thus, the present thermoresponsive solid dispersion represents a solvent-free, reduced thermal input approach compared to alternative conventional solid-dispersion techniques.

## 4. Conclusions

In the present study, a thermoresponsive solid dispersion was developed and optimized to address pharmaceutical solubility challenges using an environmentally friendly approach. The systematic Stat-Ease 360 optimization yielded a formulation comprising 10.07% *w*/*w* Pluronic F-68 and 9.98% *w*/*w* atorvastatin calcium that achieved dissolution enhancement. It was found that an optimized thermoresponsive solid dispersion improves dissolution at 5 min from 13.8% to 96.6% compared to the raw drug. The optimized system exhibits a physiological trigger performance, with a phase transition temperature of 32.00 °C and a phase transition interval of 111.66 s, remaining solid at storage temperature under appropriately controlled conditions and triggering rapidly in response to physiological conditions. The industrial manufacturing assessment showed that it offers a solvent-free alternative to conventional methods by eliminating the use of organic solvents and reducing energy consumption. The technology also offers simplified equipment requirements. As this proof-of-concept study evaluated a single model drug, further work is required to assess the applicability of the approach to other poorly water-soluble drugs and to characterize its physical stability and scalability.

## Figures and Tables

**Table 1:** Suggested thermoresponsive solid dispersions by Stat-Ease 360 software.

Std	Run	Pluronic F-68 Concentration (% *w*/*w*)	Atorvastatin Calcium Concentration (% *w*/*w*)
3	1	5	10
4	2	15	10
11	3	10	7.5
5	4	5	7.5
13	5	10	7.5
8	6	10	10
7	7	10	5
12	8	10	7.5
10	9	10	7.5
6	10	15	7.5
2	11	15	5
9	12	10	7.5
1	13	5	5

**Table 2:** Results of the measured responses for the suggested thermoresponsive solid dispersions.

Run	Phase Transition Temperature (°C)	Phase Transition Interval (Seconds)
1	29	69
2	32.5	143
3	32	110
4	30.5	78
5	32	113
6	31.5	104
7	32.5	126
8	32.5	122
9	32.5	118
10	33.5	157
11	33.5	153
12	32.5	103
13	31.5	73

**Table 3:** Analysis of variance and fit statistics for the models selected for the measured responses.

Responses	Selected Model	R^2^	Adjusted R^2^	Predicted R^2^	Adequate Precision	*p*-Value
Phase transition temperature (°C)	Quadratic	0.9707	0.9498	0.8523	23.5799	<0.0001
Phase transition interval (seconds)	Linear	0.9588	0.9506	0.9311	29.5932	<0.0001

**Table 4:** Analysis of variance (*p*-values) of the model terms for the phase transition temperature and phase transition interval.

Source	Phase Transition Temperature	Phase Transition Interval
Pluronic F-68 concentration	<0.0001	<0.0001
Atorvastatin calcium concentration	0.0003	0.0420
Pluronic F-68 × atorvastatin calcium (interaction)	0.0276	—
Pluronic F-68 concentration (quadratic)	0.0777	—
Atorvastatin calcium concentration (quadratic)	0.0777	—

**Table 5:** Predicted versus actual values of phase transition temperature and phase transition interval for optimized thermoresponsive solid dispersion.

Response	Predicted Mean	Actual Mean *
Phase transition temperature (°C)	31.26	32.00 ± 0.00
Phase transition interval (seconds)	107.58	111.66 ± 1.53

* mean ± SD (n = 3).

**Table 6:** Comparison of the process characteristics of conventional solid-dispersion techniques reported for atorvastatin calcium with the present thermoresponsive solid dispersion.

Preparation Method	Organic Solvent	Thermal/Energy Input	Unit Operations	Post-Processing (Milling/Sieving)	Principal Process Consideration	References
Spray drying	Required	Heated drying gas + pneumatic atomization	Multiple	May be required	Organic-solvent handling; specialized equipment	[13,35,36]
Solvent evaporation	Required	Heating + reduced-pressure solvent removal	Multiple	May be required	Organic-solvent handling; batch solvent-removal step	[37,38]
Freeze-drying (lyophilization)	Not required	Freezing + prolonged vacuum drying	Multiple	May be required	Long processing time; high energy demand	[39]
Hot-melt extrusion	Not required	Barrel heating + mechanical shear	Multiple	Required	Thermal/shear stress; downstream processing	[40,41]
Microwave-induced	Not required	Microwave irradiation	Multiple	May be required	Downstream processing	[42]
Fusion/melting	Not required	Carrier melting + cooling	Multiple	May be required	Thermal exposure; downstream processing	[43,44]
Thermoresponsive solid dispersion	Not required	Mild warming (40 °C) + cooling	Single vessel	Not required (solidifies in capsule)	Solvent-free, low-energy, single vessel preparation	This study

## Data Availability

The original contributions presented in this study are included in the article. Further inquiries can be directed to the corresponding author.
